# Supplementary material for: Unveiling the Synergy of Coupled Gold Nanoparticles and J-Aggregates in Plexcitonic Systems for Enhanced Photochemical Applications
Source: Nanomaterials (Basel). 2023 Dec 22;14(1):35. doi: 10.3390/nano14010035 (PMC10780452; doi:10.3390/nano14010035)
Supplement: Supplementary file 1 [file nanomaterials-14-00035-s001.zip › nanomaterials-2750513-supplementary.pdf]

Supplementary Materials: Unveiling the Synergy of Coupled Gold Nanoparticles and J-Aggregates in Plexcitonic Systems for Enhanced Photochemical Applications

Alba Jumbo-Nogales[1], Anish Rao[1], Adam Olejniczak[1], Marek Grzelczak [1,2] and Yury Rakovich [1,2,3,4,*]

S1. S2275 dye molecule.

The dye molecule known as S2275 was characterized by the UV-vis and PL spectra to establish the monomeric and J-aggregates response.

S1.1. S2275 monomeric absorbance and PL response.

The monomeric response of this dye is manifested when it is diluted in methanol, using a 10 μM solution. Figure S1 shows the absorbance and PL response coming from the monomer. The maxima of both responses (λ_{max}^{abs} = 566 nm and λ_{max}^{PL} = 582 nm) are separated by a 14 nm Stokes shift.

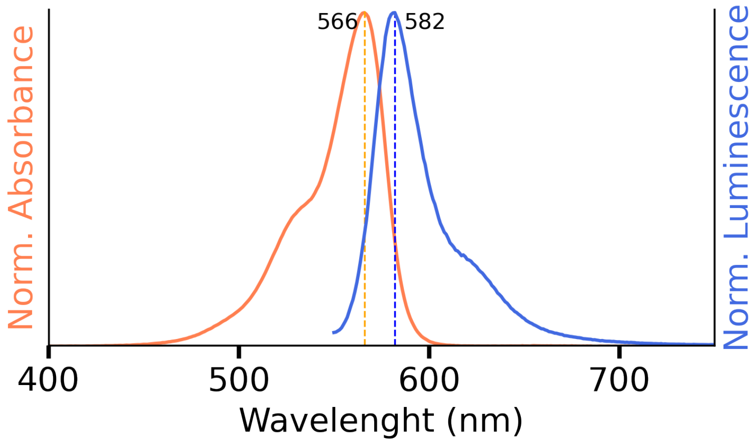

Figure S1. Monomer solution characterization. The absorbance spectrum (orange line) and the PL spectrum (blue), are separated by the Stokes shift. The emission response was obtained using a 550 nm excitation.

The J-aggregates of this dye can be obtained when they are dissolved in a NaCl aqueous solution. A dye concentration of 50 μM and NaCl 300 mM was used to acquire the J-aggregates response presented in Figure S2. The J-band is narrower and red-shifted with respect to the monomeric response, it is located at 651 nm (absorbance) and 662 nm (PL). The Stokes shift is 11 nm, shorter than the one shown by the monomer solution.

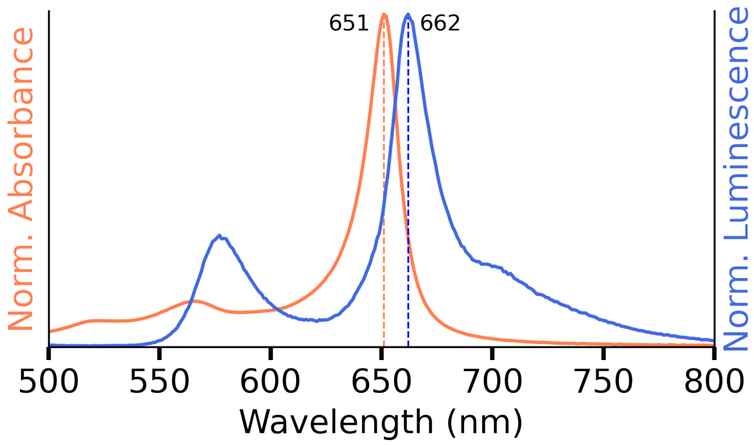

Figure S2. Dye J-aggregates characterization. The absorbance (orange line) and PL (blue line) responses of J-aggregates reflect the narrower and shifted band.

S1.2. Sodium Chloride effect in the dye J-aggregates.

The effect of NaCl on the response of J-aggregates (absorption at 650 nm) was analyzed running two experiments. First, we performed an experiment to observe how the J-band grows by raising the NaCl concentration in the solution. The maximum of the J-band was plotted with respect to [NaCl]. A sigmoidal fitting was applied to estimate the minimum NaCl concentration required to obtain J-aggregates of S2275 dye (10 μM): $\sim$97.4 mM. This can be seen in Figure S3 (**a**).

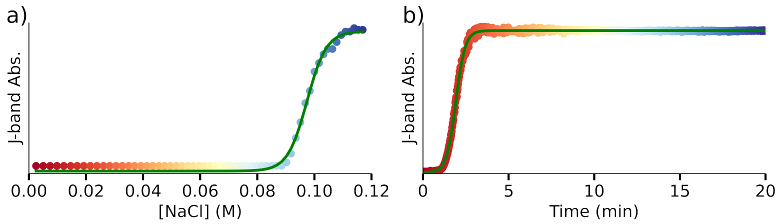

Figure S3. J-band evolution with NaCl concentration and time. (**a**) The J-band maxima value (colored dots) shows the J-aggregates formation with increasing NaCl concentration in the solution, and the green line shows the corresponding sigmoidal fitting. (**b**) The J-band evolution with time (colored dots) makes visible the J-aggregates formation with NaCl fixed concentration. The green line corresponds to the sigmoidal fitting.

Then, we inspected the time evolution of J-band under constant dye (35 μM) and NaCl (100 mM). Using a sigmoidal fitting (Figure S3 (**b**)) we were able to determine that J-aggregates require at least 2 minutes to form. Also, this analysis allowed us to estimate J-aggregates' rate of formation: $\sim$ 3.6 min^{-1}.

S1.3. J-aggregates optical image.

The optical image of the J-aggregates solution allows us to observe the elongated morphology formed by the aggregated structures. Figure S4 presents the acquired image, where the blue-colored aggregate structures can be observed.

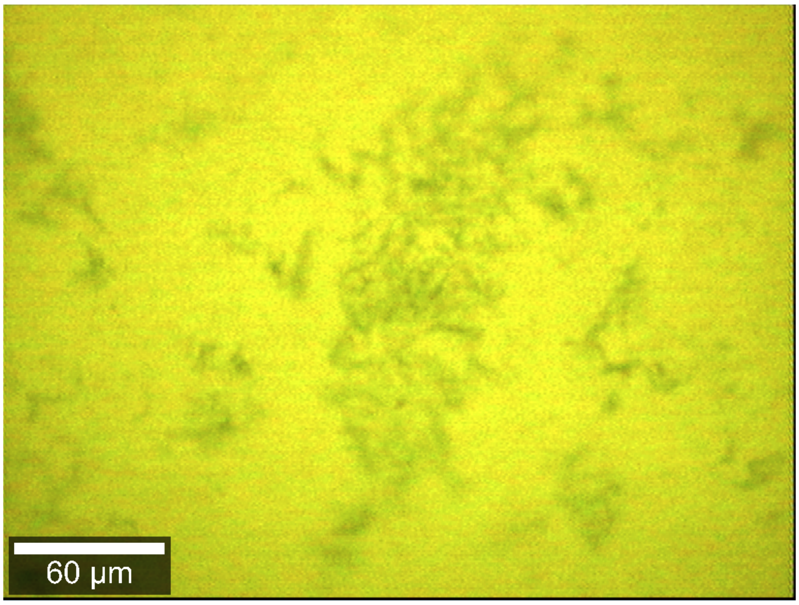

Figure S4. Bright field image of a J-aggregates solution.

S1.4. CTAB interaction.

The dye J-aggregates formation was tested in CTAB 1 mM solution. A high dye concentration (at least 80 μM) is required to observe the J-band response. For this, we used 10 mM NaCl concentration in the 1 mM CTAB solution. Figure S5 (**a**) shows the J-band formation in time after the NaCl addition. In part S5 (**b**), the evolution of the J-band evolution with time is presented. It must be remarked that the J-band maxima is reached after around 6.5 hours ($\sim$ 400 min).

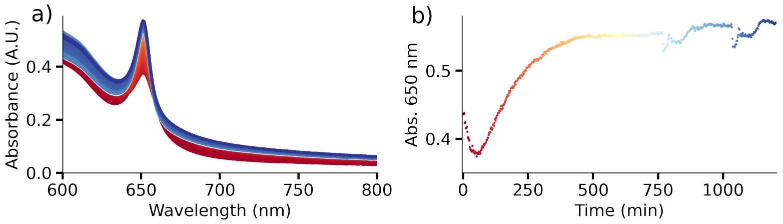

Figure S5. JC1 J-aggregates in CTAB: (**a**) absorbance spectra evolution of J-aggregates response after NaCl add. The rise of the J-band (**b**) shows how the maxima progress with time.

S2. Plexcitonic system.

The main goal of this work is based on the construction of a plexcitonic system based in AuBPs and J-aggregates.

S2.1. AuBPs capped with CTAB.

Since it is possible to form J-aggregates in CTAB solution, we proceed with the plexcitonic system formation using AuBPs in CTAB. In Figure S6 (**a**) the response of the J-aggregates in an AuBPs CTAB 1 mM solution is summarized using the absorbance spectra. We required 80 μM dye concentration in a 2 mL solution of [Au^0] = 0.5 mM AuBPs. After this, we added NaCl to obtain 10 mM concentration, under magnetic stirring. The spectrum taken (orange line) shows no clear signature of J-aggregates, possibly due to their low concentration. After this, we centrifuged our sample but just AuBPs response was observed and the dye was removed. In the second attempt, we decided to add a concentrated solution of AuBPs in CTAB (300 μL) to an aqueous solution of J-aggregates and salt solution to obtain [Au^0] = 0.5 mM and 80 μM dye. Figure S6 (**b**) shows the obtained results. In this case, the J-aggregates response can be appreciated superposing the AuBPs

spectrum. Particle aggregation was also observed. After centrifuge, the system showed a decreasing response from the J-aggregates manifesting that the system was separated.

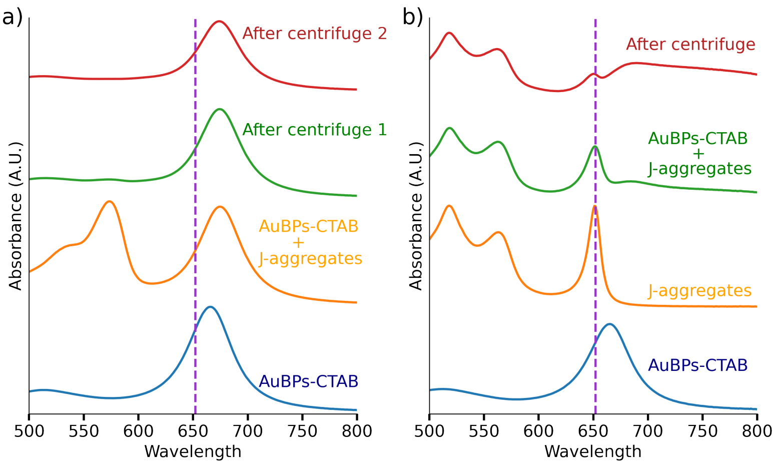

Figure S6. AuBPs in CTAB 1 mM and J-aggregates interaction. (**a**) The absorbance spectrum of AuBPs in CTAB solution (blue line) marks the initial response, then we added the dye (orange line) resulting in a strong monomeric response but there is no J-band signature. To purify our sample from the monomer molecule and enhance a possible J-band response, we proceeded to centrifuge the sample. The spectrum after this process (green line) shows the monomer removal but J-aggregates are not present even after a second purification. (**b**) The second possibility to construct the plexcitonic system involved the addition of the AuBPs (blue line) to the J-aggregates solution (orange line). The spectrum shows the J-aggregates response superposed to the AuBPs (green line). In the purified system spectra (red line) we can appreciate that J-aggregates are in large part removed, indicating that no plexcitonic system was formed.

S2.2. Systems deposited on glass.

A layer of J-aggregates was deposited on a previously functionalized glass substrate. The UV-Vis-NIR spectra show the response from the molecular chains in solution (blue line) and when they are deposited in the substrate in Figure S7. The J-band presents the same maxima in both cases, but this response is broader for the deposited J-aggregates.

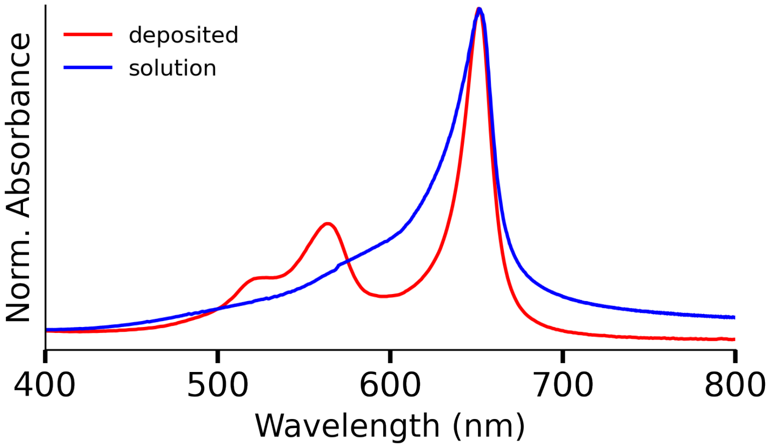

Figure S7. J-aggregates response in solution and deposited on a glass substrate. The blue line shows the absorbance response measured from the J-aggregates solution, and the red one comes from the deposited layer on a glass substrate.

To analyze the behavior of a dried sample, we also deposited the hybrid system on a glass substrate. The absorbance response was measured to prove the presence of the

plexcitonic system. Figure S8 shows the UV-vis spectra of the hybrid system in solution (blue line) and the one obtained from the deposited layer (red line). The doublet structure can be appreciated in both spectra, and in the deposited system it is red-shifted due to the media change (from water to air).

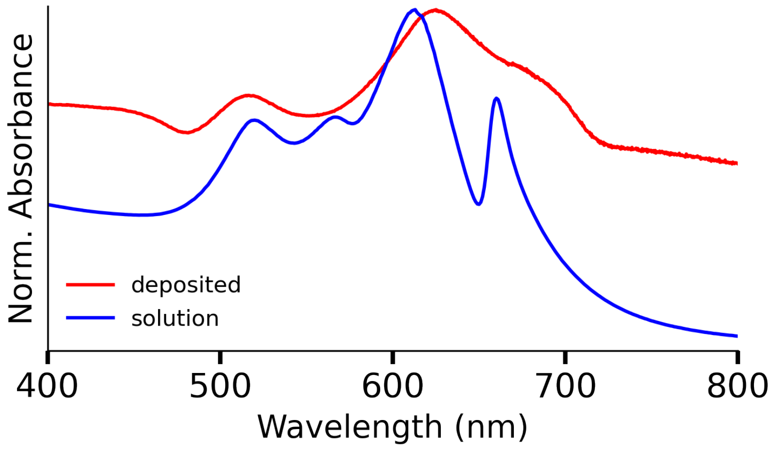

Figure S8. Plexcitonic system absorbance response in solution and from a deposited layer. The blue line shows the absorbance response measured from the hybrid systems solution, and the red one presents the spectra obtained from the system-deposited layer on a glass substrate.

S2.3. Dye PL lifetimes.

The PL decay of the S2275 dye was analyzed by estimating the monomeric and J-aggregate lifetimes. A one-exponential fitting was used for monomer decay and a bi-exponential function for J-band decay. The obtained values are presented in Table S1. The prevalence of τ_1 is shown through A_1 parameter.

Table S1. Estimated lifetimes of monomer and J-aggregates dye.

**Sample**	τ_1 **(ns)**	A_1 (%)	τ_2 **(ns)**	A_2 %	τ_{av} **(ns)**
Monomer	0.3	-	-	-	0.3
J-aggregates	0.2	99.7	2.3	0.3	0.4

S2.4. Plexcitonic system PL lifetimes.

The photoluminescence intensity decays were measured for the samples presented in this work in solution. In Table S2, these results are summarized. A bi-exponential fitting was applied to the obtained decay curves to estimate the lifetime components. A shorter $\tau_1 2$ component was found (2.3 ns) and the A_2 parameter shows a notably higher value (in almost all samples) in comparison to bare J-aggregates results.

Table S2. Estimated lifetimes of hybrid systems and their corresponding coefficients.

**AuBPs LSPR (nm)**	τ_1 **(ns)**	A_1 (%)	τ_2 **(ns)**	A_2 %	τ_{av} **(ns)**
612	0.3	98.3	1.8	1.7	0.4
635	0.2	98.6	1.3	1.4	0.4
638	0.3	99.6	1.7	0.4	0.4
641	0.3	99.4	1.7	0.6	0.3
683	0.2	97.3	1.5	2.7	0.9
700	0.3	97.4	1.8	2.6	0.5

S3. Light irradiation details.

The UV-Vis-NIR spectrum of the light used to probe the photosensitivity of J-aggregates is presented in Figure S9.

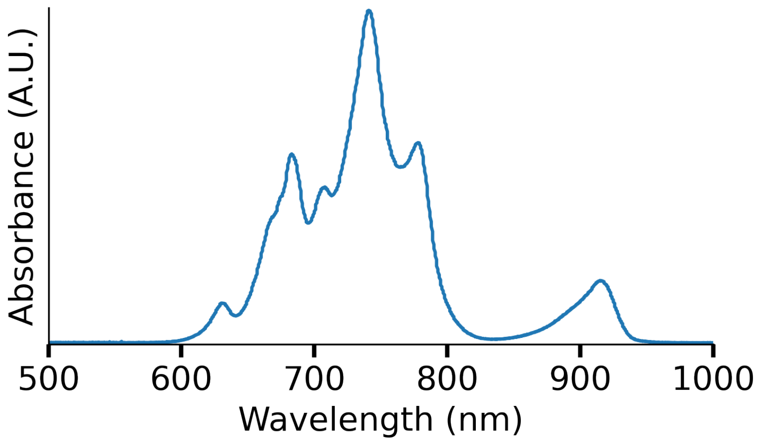

Figure S9. Used light spectrum from the G2V pico solar simulator.
